# The Emerging Role of Nanoparticles Combined with Either Radiotherapy or Hyperthermia in Head and Neck Cancer: A Current Review

**DOI:** 10.3390/cancers17050899

**Published:** 2025-03-06

**Authors:** Elena Vlastou, Andromachi Kougioumtzopoulou, Kalliopi Platoni, Ioannis Georgakopoulos, Nefeli Lagopati, Vasileios Kouloulias, Anna Zygogianni

**Affiliations:** 1 Radiotherapy Department, General Children’s Hospital ‘Pan. & Aglaia Kyriakou’, 11527 Athens, Greece; elenabls@med.uoa.gr; 2 Department of Clinical Radiation Oncology, “ATTIKON” General University Hospital, Medical School, National and Kapodistrian University of Athens, 12462 Haidari, Greece; andromachi.kou@gmail.com (A.K.); kplatoni@med.uoa.gr (K.P.); 3 Radiotherapy Unit, 1st Radiology Department, ‘Aretaieion’ University Hospital, Medical School, National and Kapodistrian University of Athens, 11528 Athens, Greece; ioangeo@med.uoa.gr (I.G.); azygogianni@med.uoa.gr (A.Z.); 4 Laboratory of Biology, Department of Basic Medical Sciences, Medical School, National and Kapodistrian University of Athens, 11527 Athens, Greece; nlagopati@med.uoa.gr

**Keywords:** radiotherapy, nanoparticles, head and neck cancer, radiosensitizers, radioenhancers, radiosensitivity, hyperthermia

## Abstract

Head and neck cancer (HNC) includes various malignancies and represents the seventh most common cancer worldwide. The early diagnosis of HNC results in a 70–90% five-year survival rate, which declines with locally advanced stages of disease. Current care employs a multimodal strategy encompassing surgery, radiation therapy (RT), chemotherapy, and immunotherapy, while treatment options vary according to the stage, tumor features, and patient characteristics. About 75% of patients with HNC will benefit from RT, either as a primary treatment or as adjuvant therapy following surgical resection. Technological improvements in RT, such as intensity-modulated RT (IMRT) and image-guided RT (IGRT), have enhanced tumor targeting and minimized adjacent healthy tissue irradiation while also expanding RT to the recurrent or metastatic setting. Innovative therapeutic strategies for HNC integrate RT with immunotherapy, gene therapy, molecular targeted therapy, photodynamic therapy, photothermal therapy, and nanoparticles (NPs), with the objective of optimizing tumor control while reducing damage to normal tissues. NPs are emerging as possible radiosensitizers in HNC treatment, enhancing the efficacy of RT, chemotherapy, and immunotherapy. In vivo and in vitro studies on the irradiation of tumors containing gold (Au), gadolinium (Gd), and hafnium oxide (HfO_2_) NPs show promising results in enhancing tumor destruction and survival rates, indicating their potential for clinical application. Hyperthermia, investigated as an adjunct treatment, potentially improves outcomes when combined with RT or chemotherapy, with advancements in nanotechnology renewing interest in this approach in HNC. At present, NBTXR3 is the sole NP that is being investigated in clinical trials for the enhancement of HNC RT.

## Simple Summary

Emerging advances in the field of nanotechnology have highlighted nanoparticles (NPs) as potential breakthroughs in the treatment of cancer. This review focuses on the possible clinical benefit of the application of NPs in head and neck cancer (HNC) radiotherapy. Preclinical studies have shown that NPs may serve as ideal radiosensitizers in radioresistant tumors like HNC. Gold and gadolinium NPs seem promising for enhancing tumor destruction and increasing survival rates. In addition, NPs have renewed scientific interest in hyperthermia for HNC. Currently, the sole nano-radioenhancer that is being investigated in the clinical setting for the optimization of HNC RT is NBTXR3.

## 1. Introduction

Head and neck cancer (HNC) encompasses a broad spectrum of malignancies arising from diverse anatomical sites, with over 90% being squamous cell carcinoma [1]. HNC represents the seventh most common cancer worldwide, with an increasing incidence seen for human papillomavirus (HPV)-associated oropharyngeal cancers [2,3,4]. The American Cancer Society reports an increasing trend of incidence of 1% and a relative mortality rate of 2% per year for HPV-related oral cancers confined to the tongue, tonsils, and oropharynx, indicating a possible 20% increase in new HNC cases by 2030 [5,6]. Alcohol and smoking still remain strong risk factors for head and neck malignancies, along with viral infections with HPV and Epstein–Barr virus (EBV) [2,7,8]. The five-year survival rate for HNC varies significantly depending on tumor location and stage; early diagnosis may yield a survival rate of 70–90%, which declines with late diagnosis [9]. Tumor and patient characteristics determine the optimal treatment approach. Currently, the management of head and neck squamous cell carcinoma (HNSCC) involves a multimodal therapeutic approach, including surgery, radiotherapy (RT), and systemic therapy such as chemotherapy and immunotherapy [10,11]. Early-stage HNSCC is treated with either surgery or definitive RT, both modalities yielding comparable rates of local control and survival. Postoperative RT with or without chemotherapy is indicated for HNSCC patients depending on the initial staging of the disease and the presence of high-risk features (lymph node involvement, positive surgical margins, extracapsular nodal invasion, perineural invasion, and vascular invasion) [12,13]. In locally advanced HNSCC, a multimodality approach incorporating surgery, RT, and chemotherapy enhances locoregional control and survival [10].

Over the last decades, technological novelties have led to the evolution of RT. Modern linear accelerators perform more advanced RT techniques, such as intensity-modulated RT (IMRT) and volumetric-modulated arc therapy (VMAT), with real-time image guidance [14,15,16]. The current RT treatment planning techniques can provide more conformal and homogeneous dose distributions, as well as steeper dose gradients. Providing superior tumor dose coverage and minimizing the radiation exposure of organs at risk (OARs) results in enhanced tumor control and improved patient quality of life (QoL) [17,18]. The integration of image-guided RT (IGRT) into IMRT or VMAT has the potential to minimize the uncertainties associated with the patient’s set-up or organ motion, improving the precision of dose delivery. In addition, pre-treatment imaging enables radiation oncologists to track any anatomic alterations occurring over the course of treatment, either to the tumor or to OARs. Monitoring anatomical changes in the target volume and OARs with IGRT throughout irradiation has resulted in the introduction of adaptive RT for treatment re-planning [19,20,21].

The evolution of RT has contributed not only to improvements in oncological outcomes but also to the broadening of indications for RT, such as re-irradiation in cases of recurrent inoperable tumors or second primary malignancies [22]. Recent studies indicate that re-irradiation using IMRT, stereotactic body RT (SBRT), and particle therapy achieves sufficient local control that can contribute to a survival benefit [23,24].

As mentioned above, RT is a common therapeutic strategy involved in HNC management. HNC’s radioresistance dictates dose prescriptions for tumor lesions from 45 up to 74 Gy, depending on tumor staging and localization. Therefore, treatment planning for HNC remains a challenge for RT professionals due to the radiosensitive OARs located in the vicinity of the target regions, such as the oral cavity, salivary glands, parotids, mandible, thyroid, spinal cord, etc., which control critical human functions (breathing, chewing, swallowing, etc.) [25,26]. Although a higher dose could improve tumor control, the tolerated dose for normal tissues lying adjacent to the tumor prohibits any further increases [27,28].

Undoubtedly, modern planning techniques have offered tremendous dosimetric advantages in HN RT [17,18,29]. However, despite the lower rate of adverse events that are reported with the utilization of modern RT techniques, RT-related toxicities to HN structures are still observed. Acute and late toxicities encompass oral mucositis, partial tongue paralysis, xerostomia, dysgeusia, dysphagia, osteoradionecrosis, skin wounds, eye damage, and thyroid dysfunction [10,30]. Apparently, further optimization of the currently implemented strategies for HNC RT that would maximize tumor control, without the induction of further damage to the surrounding normal tissues, could broaden the therapeutic window of RT and enhance patient QoL.

Hyperthermia (HT) is a form of physical therapy that employs the biological thermal effect and physical properties of non-ionizing radiation to elevate tissue temperature, thereby inducing tumor cell death or facilitating tumor cell apoptosis, since it is well established that treatment at temperatures within 40–44 °C can lead to cytotoxic effects within the tumor microenvironment [31,32]. Furthermore, HT is considered one of the most efficient radiosensitizers, as it induces DNA damage repair inhibition and the sensitization of cells in the S phase of the cell cycle and of hypoxic cells [33]. The positive role of hyperthermia is well known since time immemorial [34]. Throughout the years of HT’s long history, there have been periods of wide use and periods of skeptical opposition to the practices that were available [35]. Additionally, HT can be locoregional or whole-body and can be further classified based on the heating modality employed [32,36]. HT has proven to cause synergistic sensitization to RT, chemotherapy, and immunotherapy, and it may serve as an adjuvant treatment to enhance their outcomes through a mechanism that is known as reoxygenation [37,38]. HT combined with RT alone for HNC improves the complete response rate by approximately 25% [39]. Recent technological advancements in accurate patient positioning, three-dimensional simulation and treatment planning, heating dosimetry, and the evolution of nanotechnology have renewed interest in HT for HNC [40,41,42].

The emerging field of nanotechnology has highlighted nanoparticles’ (NPs’) potential in HNC treatment due to their unique physicochemical properties. Owing to their small size, NPs can be passively delivered to cancerous cells through the enhanced permeability and retention (EPR) effect, while active targeted delivery could be achieved through the functionalization of NPs with aptamers, receptor-specific peptides, and monoclonal antibodies. Surface modification strategies could also be employed to obtain the desired properties (biocompatibility, preferential uptake from cells, drug delivery, gene therapy, etc.). Numerous in vitro studies focusing on HNC cell irradiation have investigated the potential use of NPs either as radiosensitizers or as carriers for chemotherapy agents and target therapy agents and, in some cases, as hyperthermia triggers [43,44,45,46,47,48,49]. Studies on irradiated HNC cell models with the addition of high-atomic-number (Z) metal NPs, such as silver (Ag), gold (Au), gadolinium (Gd), and metal oxide NPs such as hafnium oxide (HfO_2_), have demonstrated increased cytotoxic effects compared to irradiation alone [44,46,47,48,49]. Preclinical studies conducted on the irradiation of HNC models indicate that the addition of AuNPs or GdNPs to tumor therapy could significantly improve the radiotherapeutic effect. So far, clinical trials on NP-aided RT in HNC patients involve solely HfO_2_ NPs.

RT holds an integral role in the treatment of HNC alone or in combination with chemoradiotherapy (CRT), while technological advances provide better treatment outcomes. Novel therapeutic approaches for HNC involve or combine RT with immunotherapy, gene therapy, molecular targeted therapy, photodynamic and photothermal therapy, and NPs in order to maximize tumor control and minimize normal tissue damage [10,11,50,51]. The aim of this work is to report the latest data from preclinical studies and clinical trials conducted in the field of NP-mediated HNC RT and HT.

## 2. NPs in RT

The rationale behind (high-Z) NP-aided RT is based on the increased Z of a tumor region upon NP accumulation. The mechanisms of (high-Z) NP-loaded tumor radiosensitization enhancement can be described by the laws that govern photon interactions with soft tissue. Photoelectric absorption is the main interaction process of kV energy photons with soft tissue (low Z). The proportionality of the photoelectric interaction probability to ~(Z/E)^3^ indicates that photons in the kV region would produce an additional cascade of secondary photons and short-range e- (i.e., Auger e^−^) upon photoelectric interactions with high-Z nanomaterials that are embedded in soft tissues. The enhanced short-range e- shower could yield an increase in dose deposition, oxidative stress, free radical generation, cell cycle disruption, and DNA repair inhibition in the NP-loaded regions. Thus, the enhancement of DNA damage and cytotoxicity would cause more effective malignant cell destruction.

**Figure 1:**
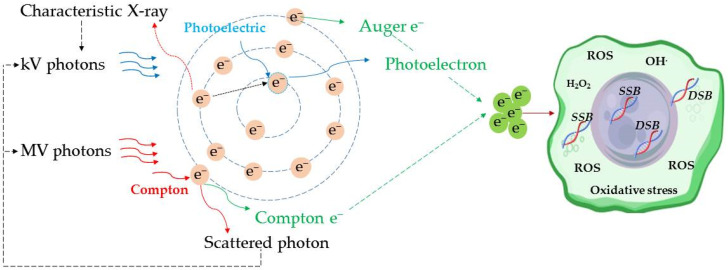
The predominant physical mechanisms of photon (energy ≤ 10 MV)–soft tissue atom interactions and their biological effects.

In the MV range, for photon energy ≤ 10 MV, Compton scattering is the dominant interaction process with soft tissue. Since the Compton cross-section has a weaker dependence on Z (proportional to Z), the irradiation of NP-loaded tissues by photon beams in the MV range would lead to lower dose enhancement. However, the typical photon beams used in external beam RT are poly-energetic; thus, the low-energy component could trigger photoelectric interactions with NPs. In addition, the low-energy photons produced by interactions between NP-loaded soft tissue and high-energy photons (by Compton effect) could also be part of the low-energy photon shower with a higher photoelectric cross-section. The predominant physical mechanism of photon–soft tissue interactions for photon energy ≤ 10 MV and its biological effect are described in Figure 1.

**Figure 2:**
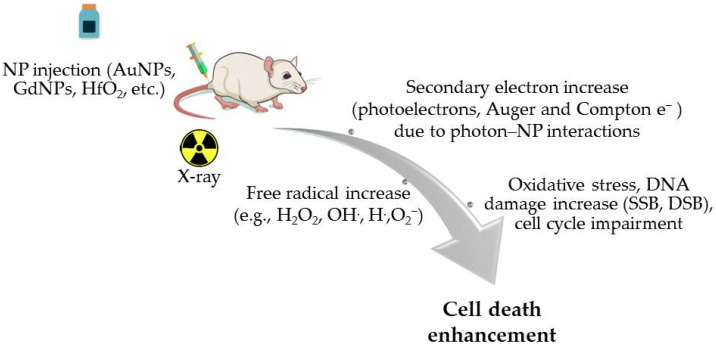
A brief representation of NPs’ radiosensitization mechanism in an animal model.

Results from studies in the literature where NP-loaded regions were irradiated using kV photons have shown a 1.1- to ~3-fold increase in dose deposition depending on the NPs’ concentration and physicochemical characteristics and the photon beam properties [52,53,54]. The majority of in silico and in vitro studies investigating the irradiation of NP-loaded regions with MV photons showed a local dose enhancement of ~1–15% depending on simulation parameters, NP concentration and size, and photon beam energy [54,55,56,57,58]. While not negligible, dose amplification in MV photon irradiation related to NP addition to targets is lower compared to kV. Nonetheless, in vitro and in vivo studies using photon irradiation in the MV range in conjunction with NPs indicate enhanced mortality rates of malignant cells, tumor growth delay, and an increase in mice’s overall survival [57,59,60,61,62]. A brief representation of NPs’ radiosensitization mechanism in an animal tumor model is presented in Figure 2.

Focusing on the oxidative stress that is the main cause of apoptosis in cancer cells upon RT in the presence of NPs, several aspects of the mechanism have been proposed. Thioredoxin reductase, a flavonoprotein (selenoprotein), is of crucial importance for cell proliferation, acting as an antioxidant barrier and participating in redox signaling [63]. Actually, thioredoxin reductase provides an important reducing equivalent to many cellular processes. The system that involves the action of thioredoxin reductase is involved in the direct regulation of several transcription factors and metabolic processes, including DNA synthesis and apoptosis [64]. Several compounds can inhibit thioredoxin reductase, such as anticancer agents, naphthazarin derivatives, organochalcogenides, iodoacetamide, etc. [65]. Using RT in parallel with the use of NPs can also inhibit thioredoxin reductase, leading cancer cells to undergo programmed cell death [66,67].

NPs, mainly metallic ones such as AuNPs, allow for targeting the areas of interest using nanocarriers that can specifically and controllably release therapeutic agents, thus maximizing the efficiency of the therapeutic approach and minimizing side effects, since the required ionizing radiation dose can be decreased upon sensitization using NPs [68]. Yet, in a case where a dose increase could offer better tumor control, the prescribed radiation dose in NP-loaded regions may not be decreased in order to enhance the absorbed tumor dose without causing any further radiation-related side effects.

## 3. NPs in HNC RT

### 3.1. In Vivo Models

#### 3.1.1. Gd NPs

AGuIX (Activation and Guidance of Irradiation by X-ray) are sub-5 nm nanoparticles that are composed of a polysiloxane matrix with Gd cyclic chelates covalently grafted on the inorganic matrix [69]. Based on preclinical data, AGuIX could serve as both radiosensitizers and MRI contrast agents [47,48,49,70]. AGuIX can preferentially accumulate in tumors without aggregating in the surrounding healthy tissues [71]. Moreover, their efficient renal clearance eliminates potential adverse events that might result from prolonged retention [72].

The use of GdNPs was investigated on CAL33-Luc (tongue carcinoma) and SQ20B (laryngeal carcinoma) orthotopic tumor models in a case where RT was delivered 42 days postoperatively [73]. Thirty-six mice were randomly assigned to three groups: the control group (10) underwent surgery alone, the RT group (13) received surgery plus postop RT, and the experimental group (13) underwent surgery plus postop RT combined with NPs. A single fraction of 10 Gy (200 keV/2 mm Al) was delivered 1 h after the intravenous injection of 200 μL of AGuIX in the experimental group (CAL33-Luc model). The median survival was 54 days for the control group, 75 days for the RT group, and 196 days for the experimental group. The survival enhancement associated with AGuIX in irradiated mice was estimated at approximately 261%.

Based on preclinical data, GdNPs have demonstrated ideal biodistribution and radiosensitizing effects owing to increased ROS production and mitochondrial alterations [74]. Miladi et al. performed in vivo experiments to confirm the radiosensitivity of GdNPs in models of human radioresistant HNSCC [48]. The intratumoral injection of 1 μmol of GdNPs to 33 mice bearing SQ20B tumors of 300–400 mm^3^ was followed by optical imaging to verify adequate GdNP uptake by the tumor. A single fraction of 10 Gy (320 kV) was delivered to a group of 21 mice. The tumor histological analysis was conducted 7 weeks after irradiation. This study demonstrated a 5- and 11-fold decrease in GdNP-loaded tumor volume compared to irradiated-only and control mice, respectively. In 3 out of 21 mice that underwent GdNP-aided RT, a complete tumor response was achieved. Cell apoptosis increased by 208% in GBN-treated and irradiated tumors compared with irradiated-only tumors (*p* < 0.001).

#### 3.1.2. AuNPs

Owing to their unique properties (biocompatibility, selective absorption by malignant cells, functionalization potential, and low toxicity), AuNPs have been explored as radioenhancers in mouse models [75]. An ultrasmall (~2 nm in diameter), biocompatible, and atomically precise nanocluster (AuNC) was developed by Jia et al. [76]. The addition of AuNCs to human esophageal squamous cancer (EC1) cells resulted in a decrease in the cell survival fraction from ~55% (irradiation alone) to 2.7% (irradiation + AuNC). The findings from in vivo experiments confirmed that combining RT + AuNC could shrink mouse tumors. More specifically, the researchers performed an intraperitoneal injection of 100 μL of AuNCs into mice, which were treated with 4 Gy X-ray irradiation 24 h later. Coupled plasma mass spectrometry prior to irradiation showed an adequate accumulation of AuNCs inside the tumor, while only some AuNCs aggregated in the bladder. The mouse tumor sizes and body weights were measured over 14 days. Finally, the tumors in the group of mice that were irradiated in the presence of AuNCs weighed about 25% and 17% of the tumors excised from mice in the irradiation-only and no-treatment groups, respectively. The 4 Gy X-ray irradiation of AuNC-loaded tumors inhibited tumor growth by 74.2% compared to irradiation alone. Moreover, mouse heart, liver, spleen, kidneys, and lungs did not exhibit any histopathological damage.

In terms of targeted therapy, AuNP-loaded irradiation in conjunction with the EGFR inhibitor CTX has been investigated in mice. In their work, Popovtzer et al. studied the effect of RT ± AuNPs ± CTX in HNSCC mouse models [77]. AuNPs (IgG- or CTX-coated) were transferred to mice bearing tumors 10 mm in diameter via a tail vein injection. Twenty-four hours after the injection, 12 mice received radiation (25 Gy) under a 6 MV photon beam originating from a flattening filter-free linac. A customized bolus of 1 cm was placed on each mouse to counteract the dose build-up effect. Depending on tumor size, the photon field sizes ranged from 1.5 × 1.5 to 2.0 × 2.0 cm^2^. No increase in the CTX-AuNP-loaded tumor volume was found at 5 weeks post-irradiation. On the contrary, tumor volume increases of 1.03, 0.4, 0.83, and 0.3 cm^3^ were observed in mice that received no treatment, irradiation alone, CTX, and CTX + irradiation, respectively. Finally, blood tests and biopsies did not indicate any AuNP-related toxicities in terms of well-being or the function of the kidneys and liver at dosages up to 6 mg of CTX-AuNPs. Likewise, Sato et al. examined the efficiency of RT ± AuNPs ± CTX in HSC-3 mouse models [78]. AuNPs of 10 nm at a concentration of 15 µg/mL were transplanted into the backs of mice that had tumor volumes of 200–300 mm^3^. Some mice received CTX as well, at a concentration of 500 µg/mL. A photon beam was delivered through an X-ray generator (1 mm Al/150 kV/5 mA), and 49 days later, all mice were sacrificed. The tumor volumes were significantly smaller in the irradiated group of CTX-injected mice in the presence of AuNPs than in the absence of AuNPs (*p* = 0.0036). No alterations were observed in the liver, kidneys, heart, or lungs of treated animals compared to control ones. The findings of this study further support the safe and efficient implementation of AuNP-driven therapy in preclinical models.

The combination of RT with the nanomedicine CYT-6091, which consists of poly-ethylene-glycol (PEG)–Tumor Necrosis factor-a (TNF)-coated AuNPs 27 nm in diameter, was investigated in HNSCC [79]. Mice with tumors with an average size of 150 mm^3^ received three fractions of 12 Gy every 3rd day (150 kVp/6.6 mA). An intravenous injection of CYT-6091 (250 μg/kg) was performed after the first two radiation doses. The results showed a 3.5-fold and 8.5-fold delay in tumor growth in the irradiated-only group of mice and the CYT + RT group, respectively.

Superior therapeutic outcomes of RT + AuNPs compared to CRT in HNC were reported in a study by Gonelli et al. [80]. In their study, the irradiation of locally advanced human papillomavirus-positive HN carcinoma combined with a novel set of nanoarchitectures (NAs) containing AuNPs (3 nm diameter) ± cisplatin was examined in vivo. The irradiated group of mice received 8 Gy in a single fraction 24 h after the injection of NAs (5 μg). On day 9, the tumor signal in mice that received RT + NA-cluster–cisplatin was almost 50% and 60% of the signal in mice of the RT ± cisplatin and NA + RT groups, respectively. The findings of this study showed complete tumor clearance in 25% of immunocompetent models, which persisted for 60 days. The authors highlight the increased efficacy of Au NAs + RT compared to CRT in oral cancer management.

#### 3.1.3. Lipid-Based NPs

Liposomes and lipid nanoparticles are lipid-based drug delivery systems that are commonly used and can be exploited in cancer treatment [81]. Focusing on HNC, Liposome-Encapsulating Double-Stranded Nucleic Acid (Poly I:C) has been used as gene therapy. Polyriboinosinic acid–polyribocytidylic acid (Poly I:C) acts as a synthetic mimic of viral double-stranded RNA, capable of inducing apoptosis in numerous cancer cells, including HN12 [82].

Aiming to disrupt the cancer cell cycle and increase their radiosensitivity, Dehghankelishadi et al. designed two different high-density lipoprotein NPs: one with simvastatin and the other with bosutinib [83,84]. The NPs’ radiosensitization effect was investigated in vitro and in syngeneic murine HNSCC models. For the in vivo approach, simvastatin (10 mg/kg) or bosutinib (20 mg/kg) NPs were administered to mice bearing tumors with volumes of 50–100 mm^3^. An irradiation of 3 Gy was performed 2 h after the injection (160 kV/25 mA). The procedure was repeated six times throughout the mice’s therapy. In both studies, tumor growth inhibition was observed in all mice that received RT after the NP injection, while no toxicities were reported. The findings from these studies suggested that simvastatin and bosutinib NP administration led to a greater decrease in tumor size, respectively, of ~51% and ~41% compared to mice that received only RT.

In the setting of concurrent CRT treatment for HNC, Bhardwaj et al. designed biodegradable gellan- and lipid-based dual nanocarriers in a hydrogel platform (PTX-CDDP-PH) to enhance site-specific delivery of both paclitaxel (PTX^®^) and cisplatin (CDDP^®^) to HNSCC [85]. Seventy-two mice bearing tumors of 100–200 mm^3^ were divided into 12 groups: control, RT only, IV PTX^®^, IV CDDP^®^, radiosensitizer-loaded hydrogel formulations (PTX-PH, CDDP-PH, PTX-CDDP-PH), dual free drugs (PTXCDDP-FD), drug-loaded dual nanoparticles without hydrogel (PTX-CDDP-NP), PTX-PH + RT, CDDP-PH + RT, and PTX-CDDP-PH + RT. Concomitant CRT was administered: five doses of formulations were injected every 3rd day (10 mg/kg PTX and 5 mg/kg CDDP); an irradiation of 3 Gy using ^60^Co was administered 24 h after the injection. The researchers monitored the tumor volumes over 30 days. Their results showed no alterations in the tumor volume in the PTX-CDDP-PH group. On the contrary, a 4- up to a 7-fold increase in tumor volume was observed in the PTX-PH, CDDP-PH, PTX-CDDP-FD, PTX-CDDP-NP, IV PTX^®^, and IV CDDP^®^ groups. The final tumor volumes were 4 times smaller in the PTX-CDDP-PH + RT group compared to RT alone. Furthermore, the observed prolonged intracellular internalization of drugs by PTX-CDDP-PH-loaded tumors compared to conventional concurrent CRT highlights the substantial advantages of nanocarriers in HNC treatment.

PTX delivery was tested upon encapsulation with radioluminescent CaWO_4_ NPs ((CWO) within protective biocompatible/biodegradable polymer capsules of polyethylene glycol–polylactic acid (PEG-PLA) [86]. Three groups of mice were designed: control, PEG-PLA/CWO NPs, and PEG-PLA/CWO/PTX NPs. Mice with tumors of 100–150 mm^3^ were injected intratumorally with 100–150 μL of solution, resulting in 10 mg of CaWO_4_ per cc of tumor (two portions every 2 days). Each group was sub-divided into irradiated and non-irradiated. The RT schedule included 4 fractions of 2 Gy. The irradiation of PEG-PLA/CWO/PTX-injected mice bearing HN31 tumors led to ~2.6 times smaller tumor volumes compared to PEG-PLA/CWO NPs + RT and RT alone. PEG-PLA/CWO/PTX NPs + RT increased mouse survival by about 10 days relative to PEG-PLA/CWO + RT. The survival increase was statistically significant relative to any other treatment (*p* < 0.001). This study further supports the extended retention of PTX inside tumors when loaded with nanoformulas, which could suggest new options for HNC patients.

All in vivo studies reporting a combination of NPs with RT for HNC are shown in Table 1.

Other organic nanomaterials, such as polypyrrole-based conducting polymers or composite materials based on photocatalysts and conducting polymers and also hybrid materials based on thermoresponsive polymers, would also provide promising alternatives against cancer [87,88,89].

## 4. NPs in HT for HNC

NPs in the setting of HT may provide a potential benefit by enabling a more accurate heating target at the tumor site compared to conventional HT, hence reducing the thermal exposure of adjacent healthy tissues and the occurrence of adverse effects. Inorganic (metal and carbon NPs) and organic NPs (porphysome and light-absorbing conductive polymers) have been investigated for HT optimization [90].

### 4.1. Inorganic NPs

Iron oxide nanoparticles, primarily Fe_3_O_4_ and its derivatives (100 nm or smaller in size), have become favored for their minimal adverse events, rapid heating capabilities, and stability [91,92]. The Fe_3_O_4_@mSiO_2_ nanocarrier was employed to regulate the release of anticancer drugs under alternating magnetic fields, demonstrating the potential for tailored drug delivery in conjunction with magnetic hyperthermia [93]. Superparamagnetic iron oxide nanoparticles (SPIONs) may be utilized independently or in conjunction with chemotherapy and immunotherapy. An in vitro study in HNSCC indicates reduced cell proliferation in reaction to elevated SPION concentrations, implying its potential application in HNC [94]. In their study, Wang et al. evaluated 22 rabbits with VX2 tumors in pyriform sinuses that were randomly assigned to either the HT group or the control group after injecting ultrasmall superparamagnetic iron oxide (USPIO) into the submucosa beside the tumor [95]. The apoptosis rate was 100% in the HT group versus 20% in the control group (*p* < 0.05). Nevertheless, iron nanoparticles necessitate elevated concentrations to attain thermal enhancement, perhaps resulting in the killing of adjacent normal cells around the tumor [96].

AuNPs are considered among the most effective hyperthermic agents in nanomaterials due to their significantly increased absorption in the near-infrared (NIR) regions [97,98]. An in vitro study by Trinidad et al. used Au-nanoshell-loaded rat macrophages, either alone or combined with human FaDu squamous cells, which convert NIR light to heat, as transport vectors for photothermal hyperthermia of tumors [99]. The findings from this study showed that the combined treatment reduced cell viability to less than 40% at the same laser power settings.

### 4.2. Organic NPs

Based on results from in vivo and in vitro studies, organic NPs seem to have the potential to serve as agents for photothermal therapies [100,101,102]. For instance, porphyrin–lipid nanovesicles (porphysomes) that are liposome-like NPs, including pyropheophorbide-conjugated phospholipids, have proven effective as multimodal theranostic agents for phototherapies and targeted drug delivery, in parallel with in vivo fluorescence, magnetic resonance, or positron emission imaging [103]. The in vivo study of Muhanna et al. in rabbit and mouse models of buccal and tongue cancer showed the superiority of porphysome-enabled photothermal therapy over surgery in terms of the radical eradication of the tumor and the regional metastatic lymph nodes [104]. The surrounding healthy structures were not affected.

## 5. Clinical Trials Involving NPs as Radioenhancers in HNC RT

ΝBTXR3, which consists of a functionalized core of HfO_2_, was the first radioenhancer to receive CE mark approval in 2019 after the completion of a phase II/III trial for the treatment of locally advanced soft tissue sarcoma [105]. The group of patients who received NBTXR3 exhibited an increased pathological complete response rate after RT compared to the RT-only group, while no treatment-related deaths were reported. NBTXR3 holds significant radiosensitization and immunomodulatory properties [106,107,108,109,110]. NBTXR3 is the sole radioenhancer now undergoing clinical trials aimed at examining the therapeutic effects of NPs in patients with HNC treated with RT.

In the phase I study (NCT01946867) investigating the safety of NBTXR3 in frail or elderly patients with locally advanced HNSCC ineligible for cisplatin treatment concurrently with RT, five centers in France and Spain participated [111]. The aim of the trial was to assess the safety of NBTXR3. The primary endpoint was to determine the recommended phase II dose (RP2D) and dose-limiting toxicities (DLTs), and the secondary endpoints were the safety, tolerability, efficacy, and feasibility of intratumoral injection of NBTXR3. Eligibility criteria included patients ≥ 65 years of age with an accessible tumor for injection, a Karnofsky score ≥ 70, and stage III or IVA disease with no distant metastases. The study design was a traditional 3 + 3 dose escalation design. The intratumoral injection of NBTXR3 was performed on day 1 under general anesthesia, and IMRT was started on day 2; all patients were scheduled to receive 70 Gy with conventional fractionation (2 Gy/fraction, in 35 fractions). A total of 19 patients were enrolled in the study, and the primary site of the tumor was either in the oral cavity (6) or the oropharynx (13). All patients received a single injection of NBTXR3, and 16 patients completed the prescribed RT regimen. The RP2D was determined at 22% baseline tumor volume, the maximum tolerated dose (MTD) was not reached, and no serious adverse events related to NBTXR3 were observed.

The dose expansion part of this clinical trial aimed to examine the recommended dose in terms of safety and preliminary efficacy [112]. The secondary endpoints included the evaluation of progression-free survival (PFS) and overall survival (OS). Up to January 2022, 56 patients were treated according to the aforementioned protocol, 44 of which were eligible for objective tumor response evaluation. A percentage of 1.3% of treatment-emergent adverse effects were attributed to either NBTXR3 or its injection. The median PFS and OS were 16.9 and 23.1 months, respectively. Finally, the objective response rate (ORR) of the injected lesion was 81.8%, a fact that reinforces the efficacy of the NBTXR3–RT combination for elderly patients with LA HNSCC.

The intratumoral administration of NPs has shown increased efficiency and reduced off-target toxicity compared to systemic administration [113,114]. The tumor microenvironment, the NP properties, and the administration process seem to be the key parameters affecting NPs’ biodistribution [114]. In April 2024, international clinical consensus guidelines were published for the intratumoral and intranodal injection of NBTXR3 in HSNCC patients using the Delphi method to facilitate reproducibility, safety, and efficacy [115]. A strong consensus was achieved, and a comprehensive set of recommendations was provided, including the volume calculation for NBTXR3, patient selection, preparation and injection procedures, and potential adverse effects. Post-injection and post-treatment follow-up are thoroughly detailed as well.

## 6. Ongoing Clinical Trials Involving NPs as Radioenhancers in HNC RT

Following NCT01946867, a phase III trial (NCT04892173), which launched on January 2022 and is to be completed by 2026, is investigating the efficacy of NBTXR3/RT ± CTX in elderly patients with locally advanced HNSCC [116]. The primary objective is the assessment of PFS, OS, ORR, safety and tolerability, and patient QoL. A single intratumoral/intranodal injection of NBTXR3 containing 33% of the Gross Tumor Volume (GTV) is followed by RT alone or combined with CTX. The RT prescription is 70 Gy in 35 fractions. Subsequent to 50 Gy dose delivery, tumor immune response biomarker and tumor size alterations are evaluated. Results from this study are still pending.

A phase Ib/II trial (NCT02901483) enrolled patients with T3-4 locally advanced HNSCC (at least 20 years old) eligible for cisplatin chemoradiation [117]. The phase Ib study intended to determine the RP2D and assess its safety. Phase II’s purpose is to estimate the rate of locoregional control at 1 year, along with the safety profile. Pharmacokinetics, ORR, PFS at 1 year, and pathological response will be evaluated as well. A single intratumoral injection of NBTXR3 (5–33% of GTV volume) was given to 19 patients (up to June 2022) 24 to 72 h prior to chemoradiation. RT planning used IMRT to deliver 70–72 Gy (2–2.12 Gy/fraction) to the tumor lesion. Once a week, a low dose of cisplatin (40 mg/m^2^) was administered to the patients. Although RP2D and MTD were not determined due to early termination of the study, the treatment scheme was found to be safe for the patients. DLTs were reported for one patient at a 10% dose level. During treatment delivery, NBTXR3 was localized in the tumor environment without dispersion to surrounding normal tissues. Further results from this study are expected.

By November 2022, 11 patients older than 18 years, eligible for anti-PD-1, with locoregional recurrent/recurrent and metastatic HNSCC, had been treated in the context of a phase I clinical trial (NCT03589339) [118]. The patients were referred for HN re-irradiation. The trial’s objectives are similar to the former mentioned ones. Evidence from this trial supports the fact that NBTXR3/stereotactic body RT (SBRT)/anti-PD-1 is associated solely with radiation-related side effects and thus could be safely and efficiently implemented. The RP2D was defined, but to date, no published data are available in the literature. The trial is expected to complete accrual by 2028.

The combination of NBTXR3 with SBRT and the monoclonal antibody pembrolizumab as a therapeutic approach for adult patients with at least two lesions of recurrent/metastatic HNSCC is being investigated in a phase II clinical trial (NCT04862455) [119]. The tumor response, safety profile, and time-to-event outcome assessment are the primary objectives of the study. Subsequently, the investigators will link the radiomics measurements with treatment outcomes and estimate biomarkers of the response in treated lesions. On day 1, a volume of 60 cm^3^ of NBTXR3 is injected into one lesion intratumorally/intranodally under image guidance. On day 3 and within 8 days of NBTXR3 injection, either SBRT every other day or hypofractionated RT once daily for 1–2 weeks is delivered to the injected lesion. Intravenous administration of pembrolizumab (duration of 30 min) is performed at the beginning of RT and is repeated every 3 weeks for up to 24 months, provided that patients remain free of disease progression and do not exhibit unacceptable toxicities. No results have been presented so far, while September 2026 is estimated as the trial’s termination date.

## 7. Conclusions and Future Directions

RT plays an essential role in the management of HNC, with its indications and applications evolving and currently attracting significant scientific interest. Even though the implementation of IMRT, VMAT, and IGRT in the radiotherapeutic strategy has improved target dose coverage and OAR sparing, locally advanced HNSCC continues to pose a challenge in RT. RT concurrent with chemotherapy is currently the optimal treatment for locally advanced HNSCC [10,51]. Further optimization of the currently implemented strategies for HNC RT should be performed, aiming at maximized tumor control and minimized normal tissue damage. The research in the field of nanotechnology conducted over the last decade has offered clinical evidence of NPs’ beneficial role in RT. NPs may serve as optimal radiosensitizers due to their potential to enhance local tumor management without any apparent additional toxicity [120]. The addition of NPs to the target volumes could lead to tumor radiosensitization enhancement without increasing dose prescriptions, which would cause further side effects on the adjacent OARs. If NPs accumulate exclusively in tumors and given that the calculated dose enhancement from various studies in the literature is restricted to short distances from NPs, OARs would not be further affected by the induced dose amplification. Moreover, recent technological advances in HT have revived scientific interest and may represent the fifth pillar of oncology, along with surgery, RT, chemotherapy, and immunotherapy.

The outcomes from the aforementioned clinical trials indicate the safe and efficient use of NBTXR3 in humans in conjunction with RT or CRT, without any severe NP-related toxicities. Current ongoing trials including NBTXR3 are exploring other directions, including RT planning, dose adjustments, efficacy in metastatic patients, and synergistic effects with immunotherapy or chemotherapy. Injection procedures and product delivery assessments will be adjusted to address the evolving requirements of these ongoing studies.

Numerous in vitro and in vivo studies demonstrated that Au and Gd NPs’ radiosensitization properties could boost HN tumor cell destruction and lead to an increased survival rate in animal models [44,47,77,78]. Moreover, their rapid renal clearance, safe biodistribution patterns, adequate accumulation in tumors, and imaging properties render them suitable agents for RT optimization in mouse models.

However, efficacy and safety evaluation remain a challenge for the use of NPs in RT. The huge variety of NPs’ characteristics (material, size, shape, surface chemistry, concentration, coating, etc.) and experimental protocols (type of administration, interval between injection and irradiation, photon beam energy, etc.) that have been investigated in the literature complicate the intercomparison of published results. In addition, the data obtained from studies concerning the dosage, radiosensitization levels, biocompatibility, and toxicity patterns of NPs in animals do not always match clinical trial results [121]. The differences between humans and animal models stress the need for thorough research in order to clarify the exact mechanisms of NPs’ biodistribution and pharmacokinetics prior to clinical translation.

Furthermore, in future studies, emphasis should be placed on NPs’ optimal physicochemical characteristic choices (e.g., material, size, shape, and surface chemistry), since these parameters control the tumor-targeting potential and affect NPs’ stability, biodistribution pattern, clearance route, and toxicity levels. Interdisciplinary research should focus on standardizing NPs’ optimal properties in order to achieve increased therapeutic gain and decreased toxic effects.

The complex processes involved in NP manufacturing and the production costs, along with the lack of formal regulations for the production and disposal of nanomaterials, are additional obstacles in scientific research [122]. Moreover, the need for consistent and reproducible NP formulations when large quantities are needed raises further challenges in clinical trial design [121].

To overcome the barriers involved in NP-driven therapy research, new approaches are being investigated. For instance, “green” methods have been developed to minimize the cost with eco-friendly behaviors [123]. The possibility of combining those processes with machine learning and optimization methods through a Multi-Criteria Decision-Making Approach for pre-synthesis steps, the Taguchi method, or other AI-based methods can promise a new era in cancer treatment, since they could be applied in order to predict the optimal characteristics of a produced material before its synthesis in order to avoid extra costs [124,125,126]. Furthermore, in silico approaches could provide a deep understanding of NP interactions with soft tissues, building a roadmap for future in vitro and in vivo studies. Particle physics algorithms at the nanometer scale and biological modeling have made huge progress over the last several years. Thus, computational studies can perform more accurate nanodosimetric calculations and address the biological effects of NP-driven RT. The involvement of human models in Monte Carlo simulations could offer great insights into the efficacy, biodistribution, and toxicity patterns of different nanoformulas in HNC RT.

In the clinical setting, to allow for accurate dose distribution evaluation in patients under RT, modern treatment planning systems should encompass NP and nanoscale dose calculations. In addition, the potential image contrast properties of NP-loaded tumors could be examined as well to improve pre-treatment image guidance. Finally, a cost–benefit analysis would provide a clear insight into whether the clinical adaptation of NPs could be an asset in HNC patients’ treatment.

HT combined with RT shows a synergistic potential for enhancing the antitumoral effect [33]. The efficacy of adding HT to HNC treatment is supported by clinical studies, although the clinical application has not yet been established [39,127,128]. Recent technological advances in patient positioning, 3D planning, and heating dosimetry, along with nanotechnology, have renewed interest in HT in HNC [42,128]. Local HT with NPs in HNC is currently being investigated in the preclinical setting with encouraging results.

NPs appear to be promising theranostic agents that could benefit HNC patients’ treatment. Despite the huge steps toward NP-aided RT for HNC, further and consistent research is required. Apparently, academia, industry, and federal regulation agencies should be encouraged to work in close collaboration to establish the meaningful and safe use of NPs in clinical practice.

## Figures and Tables

**Table 1:** In vivo studies that combined NPs with RT in HNC.

Study (Year)	Cancer Type	NP	RT Regimen	Results
Quatre, et al. (2019) [73]	CAL33-Luc (tongue)	AGuIX (200 μL)	A single fraction of 10 Gy (200 keV)	The combination of AGuIX with RT increased survival by 261% compared to RT alone. Median survival time in a. RT only: 75 days; b. RT + AGuIX: 196 days.
Miladi, et al. (2015) [48]	SQ20B (laryngeal HNSCC after RT)	Gd (1 μmol)	A single fraction of 10 Gy (320 keV)	The combination of RT + NPs delayed tumor growth with an increase in late apoptosis and a decrease in cell proliferation: a. 5- and 11-fold decreases in GdNP-loaded tumor volume compared to RT-only and control mice, respectively; b. Cell apoptosis increased by 208% compared with irradiated-only tumors.
Popovtzer, et al. (2016) [77]	HNSCC	CTX-coated AuNPs (5 μL)	A single fraction of 25 Gy (6 MV)	The combination treatment (AuNPs + CTX + RT) was related to earlier and greater apoptosis angiogenesis inhibition and a diminished repair mechanism. It was the sole treatment resulting in no tumor growth over 7 weeks.
Sato, et al. (2023) [78]	HSC-3 (tongue HNSCC)	AuNPs (15 µg/mL) + CTX	A single fraction of 4 Gy (150 kV)	The combination treatment (AuNPs + CTX + RT) significantly reduced the tumor volume compared to CTX + RT. AuNPs had no toxic effects on normal tissues.
Koonce, et al. (2015) [79]	SCCVII (murine squamous cell carcinoma)	CYT-6091 (polyethylene glycol–TNF-coated AuNPs) (200 μg/kg)	3 fractions of 12 Gy every 3rd day (150 kV)	RT + CYT induced an 8.5-fold delay in tumor growth compared to RT only.
Gonnelli, et al. (2024) [80]	HPV16 E7-expressing TC-1/Luc cells (mimic HPV-HNSCC)	NA-cluster-CisPt (AuNPs+ cisplatin) (5 μg)	A single fraction of 8 Gy (200 kV)	The NA-cluster-CisPt + RT had significant tumor signal reduction compared to any other treatment.
Dehghankelishadi et al. (2022) [83]	MOC-1 (mouse oral squamous cell carcinoma)	HDL-NPs+ simvastatin (6 doses of 10 μg/kg)	6 fractions of 3 Gy (160 kV)	The combination therapy led to 51% smaller tumor volumes compared to RT alone. No toxicities were reported.
Dehghankelishadi et al. (2023) [84]	MOC-1 (mouse oral squamous cell carcinoma)	HDL-NPs+ bosutinib (6 doses of 20 mg/kg)	6 fractions of 3 Gy (160 kV)	The combination therapy led to 41% smaller tumor volumes compared to RT alone. No toxicities were reported.
Bhardwaz, et al. (2022) [85]	AW8507 (oropharyngeal HNCC)	Gellan- and lipid-based dual NPs + paclitaxel (10 mg/kg) or +cisplatin (5 mg/kg)	6 fractions of 3 Gy each (twice in a week) (^60^Co)	The combination therapy marked a significantly pronounced tumor-growth-inhibitory effect compared to any other treatment.
Misra, et al. (2019) [86]	HN31 (metastatic lymph node squamous cell carcinoma of the pharynx)	PEG-PLA-coated CWO NPs (10 mg per cc tumor) ± paclitaxel (1.2 mg of PTX per cc tumor)	4 fractions of 2 Gy (320 kV)	The combination therapy significantly increased mouse survival time.

Abbreviations: HNSCC, head and neck squamous cancer cell; Gd, gadolinium; NPs, nanoparticles; RT, radiotherapy; Au, gold; HDL, high-density lipoprotein; PEG-PLA, poly(ethylene glycol-b-D,L-lactic acid); CWO, CaWO_4_.
